# Memantine for the treatment of obsessive-compulsive disorder: a systematic review and narrative synthesis

**DOI:** 10.1186/s12888-026-07787-7

**Published:** 2026-01-10

**Authors:** Joshua W. Bryan, Athanasios Hassoulas

**Affiliations:** Cardiff University School of Medicine, Cardiff, UK

**Keywords:** Memantine, Obsessive-compulsive disorder, Psychopharmacology

## Abstract

**Objective:**

Obsessive-compulsive disorder (OCD) is a chronic psychiatric condition for which a substantial proportion of patients do not respond adequately to first-line treatments. This review thus aimed to critically appraise the clinical literature examining memantine in obsessive-compulsive disorder, with a particular focus on individual study design, dosing strategies, tolerability, and methodological limitations.

**Methods:**

A structured literature search of MEDLINE was conducted in March 2024 from database inception to March 2024. Titles, abstracts, and full texts were screened against predefined inclusion criteria, and relevant studies were synthesised narratively. A second, identical literature search was conducted in August 2025 covering March 2024 to August 2025. In total, 10 studies were included in the narrative synthesis.

**Results:**

Our findings suggest that memantine may offer therapeutic benefits for OCD. Methodological issues, however, such as small sample sizes, strong geographical clustering, the exclusion of dropout data and limited use of intention-to-treat analyses, restrict the generalisability of the reported outcomes. Tolerability varied significantly by dose, with higher doses being associated with increased side effects while lower doses appeared better tolerated but elicited a poorer efficacy, although evidence was mixed. Only one study rigorously assessed treatment-refractory OCD and reported significant symptomatic improvement following longer-term memantine administration. Baseline severity, treatment expectancy, and concurrent cognitive-behavioural therapy were also identified as factors that may mediate these effects.

**Conclusion:**

Overall, current evidence does not support the routine use of memantine for OCD. However, it does highlight specific methodological considerations and priorities for future rigorous investigation. Future randomised controlled trials with larger samples, longer follow-ups, and standardised dosing protocols are needed to clearly determine memantine’s role in OCD management.

**Clinical trial number:**

Not applicable.

## Introduction

Obsessive-Compulsive Disorder (OCD) is a chronic and debilitating psychiatric disorder that affects approximately 2–3% of the global population [1, 2]. It is characterised by recurrent thoughts (obsessions) and behaviours (compulsions) that cause significant distress and interfere with daily functioning [3, 4]. Selective-serotonin reuptake inhibitors (SSRIs) and exposure and response prevention (ERP) remain first-line treatments for OCD [5, 6]. Concerningly, however, approximately 20% of patients are refractory to these interventions, with full remission being uncommon [4, 7]. This highlights the vital need in identifying alternative treatment modalities.

Glutamate dysregulation in cortico-striatal-thalamocortical circuitry is well documented in OCD, with elevated glutamate levels and hyperactivity in the orbitofrontal cortex (OFC) and anterior cingulate cortex (ACC) being shown to correlate with symptom severity [8–10]. Evidence has suggested the role of adjunctive glutamate-modulating drugs in reducing OCD’s obsessive and compulsive symptoms [11, 12]. Memantine, a non-competitive N-methyl-D-Aspartate (NMDA) receptor antagonist, inflects activation of the NMDA receptor, blocking excessive excitatory activity [13] and restoring adaptive glutamate balance [14]. Whilst already approved as for use in Alzheimer’s disease [15], several studies have highlighted its potential as an adjunctive treatment in reducing the symptoms of OCD.

However, discrepancies within the literature arise from extensive variation in study protocols, including baseline symptom severity, adjunctive dosing procedures, and broader methodological limitations that have restricted the development of a coherent evidence base. In light of these discrepancies, this review aims to critically appraise the existing clinical literature examining the role of memantine in OCD, with particular emphasis on study design, dosing strategies, tolerability, and methodological limitations that influence interpretation of reported outcomes.

## Methods

### Search strategy

A structured literature search was conducted in MEDLINE using the search terms “memantine,” “obsessive-compulsive disorder,” and “glutamat*” combined with Boolean operators. The search included all available records from database inception until March 2024. An updated search was performed in August 2025 employing the same search strategy, inclusion and exclusion criteria to search for studies published since the original search.

Backward citation searching was conducted by screening the reference lists of included studies and relevant reviews. Forward citation searching was also performed to identify additional potentially eligible studies. Citation searching identified no additional relevant studies.

### Screening and selection

Titles and abstracts were screened by the author to identify potentially relevant studies, followed by full-text screening against predefined inclusion criteria. Screening was performed independently without a second reviewer, which is noted as a methodological limitation. All screening and study selection procedures were conducted by the same author using an identical set of inclusion and exclusion criteria across both searches; no changes were made to eligibility criteria between search stages.

### Inclusion and exclusion criteria

Studies were included based on the following criteria, structured according to the PICO framework:

#### Population

Individuals diagnosed with OCD, irrespective of treatment resistance status.

#### Intervention

Administration of any dose of memantine over any treatment period.

#### Comparator

Placebo or standard care controls where applicable.

#### Outcomes

Primary outcomes were changes in obsessive-compulsive symptom severity (e.g. Yale-Brown Obsessive Compulsive Scale). Secondary outcomes included cognitive or executive function (e.g. NOCC, WCST), and measures of tolerability or adverse effects. Studies were not excluded based on the reporting measure they used.

Exclusion criteria included animal studies, studies without a primary focus on memantine, case studies, and non-English language publications.

### Data synthesis

Due to the heterogeneity of the included studies in terms of study design, populations, dosing protocols and outcome measures a narrative synthesis was conducted, which precluded a meta-analysis.

The updated search conducted in August 2025 identified one additional eligible randomised controlled trial. In total, ten studies were included in the review, encompassing 450 individual participants.

## Results

### Study selection

#### First search (March 2024)

A total of 42 records were identified through the database search, and all titles and abstracts were screened. Of these, 12 full-text articles were assessed for eligibility based on the inclusion and exclusion criteria.

After full-text screening, nine studies were included in the review.

#### Second search (August 2025)

A total of five records were identified through the database search conducted from the start of 2024 to the present day, and all five titles and abstracts were screened. Of these, one full-text article was assessed for eligibility.

After full-text screening, the one additional study was included in the final review.

**Fig. 1:**
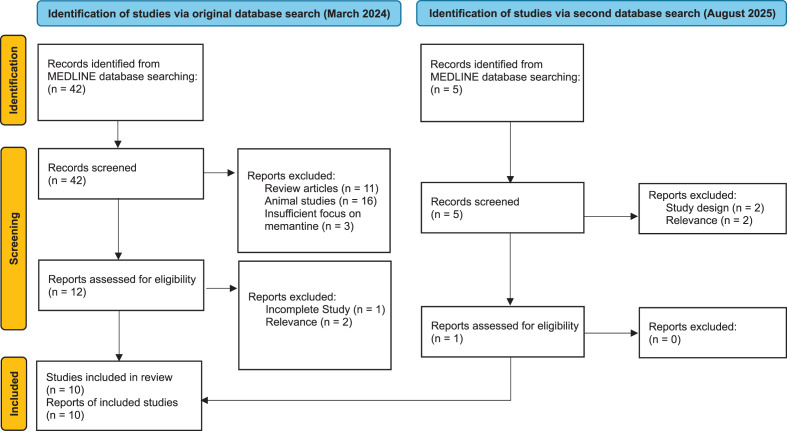
Flow diagram of study selection for the systematic review, incorporating results from two database searches (March 2024 and August 2025), following PRISMA 2020 guidelines

The study selection process is summarised in the PRISMA flow diagram (Fig. 1).

### Study characteristics

The included studies were published between 2009 and 2025, with sample sizes ranging from 10 to 99. All studies investigated the efficacy of memantine as an adjunctive treatment of obsessive-compulsive disorder. The characteristics of the included studies are summarised in Table 1.

**Table 1:** Characteristics of the included studies

Study (Author, Year)	Country	Study Design	Sample Size (N)	Population Characteristics	Intervention Details	Comparator	Outcome Measures	Duration of Follow-up	Key Findings
Aboujaoude et al. 2009 [16]	USA	Open-label trial	15	Adults with treatment-resistant OCD, baseline Y-BOCS ≥18, failed ≥1 SRI trials	Memantine (target 20 mg/day) added to current treatment	None	Y-BOCS, CGI-I	12 weeks	43% responders with ≥25% Y-BOCS reduction; mild, transient side effects; small sample and uncontrolled.
Askari et al. 2022 [17]	Iran	RCT	70	OCD patients (DSM-5), Y-BOCS > 21	Memantine (20 mg/day) + sertraline (100–200 mg/day)	Placebo + sertraline	Y-BOCS; WCST	12 weeks	No significant symptom improvement with memantine vs. placebo on Y-BOCS; memantine improved executive function (WCST); well tolerated.
Farnia et al. 2018 [18]	Iran	RCT	99	Adults with OCD (mean age 29.6, 49.5% female)	Fluoxetine (SSRI) + gabapentin or memantine (Weeks 1–4: 5 mg/day; Weeks 5–8: 10 mg/day	Fluoxetine + placebo	Y-BOCS	8 weeks	No significant improvements with gabapentin or memantine over placebo; only the placebo group showed significant improvement. Side effects were noted.
Feusner et al. 2009 [3]	USA	Open-label trial	17 (10 OCD; 7 GAD)	Adults with OCD (*n* = 10) and GAD (*n* = 7); mixed treatment status	Memantine 20 mg/day, as monotherapy or augmentation	None	Y-BOCS; HARS; CGI-I	12 weeks	Significant reduction in OCD symptoms (40.6% mean Y-BOCS decrease); limited effect in GAD. Memantine well tolerated with no serious adverse events.
Ghaleiha et al. 2013 [11]	Iran	RCT	42	Patients with moderate to severe OCD, Y-BOCS ≥ 21	Memantine add-on to fluvoxamine (Week 1: 10 mg/day; Week 2: 20 mg/day)	Placebo + fluvoxamine	Y-BOCS	8 weeks	Memantine + fluvoxamine significantly improved OCD symptoms compared to placebo; higher remission rate and well tolerated.
Haghighi et al. 2013 [19]	Iran	RCT	40	Inpatients with OCD (mean age 31.25; 80% female)	SSRI or clomipramine + memantine (5–10 mg/day))	SSRI or clomipramine + placebo	Y-BOCS; CGI-Severity; CGI-I; SGOT/SGPT (liver enzymes)	12 weeks	Memantine group showed significantly greater reduction in OCD symptoms and illness severity; no significant difference in CGI-Improvement.
Mirzazadeh et al. 2025 [20]	Iran	RCT	60	Adults with OCD	Escitalopram + memantine (10 mg/day)	Escitalopram + placebo	Y-BOCS; BDEFS; Adverse Event Questionnaire	16 weeks	Both groups had significant OCD symptom reduction; no group difference in Y-BOCS; memantine improved time management domain of executive function. Minimal side effects.
Modarresi et al. 2017 [12]	Iran	RCT	32	Patients with severe, SRI-refractory OCD	Memantine (20 mg/day) + SRI	Placebo + SRI	Y-BOCS	12 weeks	Significant improvement in OCD symptoms seen in memantine group after 8–12 weeks; 73.3% achieved treatment response. Well tolerated.
Sahraian et al. 2017 [21]	Iran	RCT	58	BD Type I patients in manic phase with OC symptoms*	Memantine (started on 5 mg/day, titrated up to 20 mg/day) + lithium + olanzapine + clonazepam	Placebo + lithium + olanzapine + clonazepam	Y-BOCS and adverse effects	16 weeks	Memantine group showed significantly greater reduction in OC symptoms (79% responders vs 37% in placebo); no serious adverse effects reported.
Stewart et al. 2010 [22]	USA	Single-blind case-control study	44	Severe OCD patients in Intensive Residential Treatment program	Memantine (5 mg/day titrated up to 18 mg/day) augmentation added to standard treatment	Matched controls receiving standard treatment only	Y-BOCS; CGIS;	Monthly assessments during IRT program	Memantine augmentation showed greater OCD symptom improvement (27% vs. 16.5%) and depression symptom reduction vs. controls; evidence supportive but RCTs needed.

Abbreviations: Y-BOCS = Yale-Brown Obsessive Compulsive Scale; CGIS = Clinical Global Impression Scale; BDEFS = Barkley Deficits in Executive Functioning Scale; SGOT/SGPT = Serum Glutamic-Oxaloacetic Transaminase/Serum Glutamic-Pyruvic Transaminase [Liver Enzymes]; CGI-Severity = Clinical Global Impression - Severity; CGI-I = Clinical Global Impression - Improvement; HARS = Hamilton Anxiety Rating Scale; WCST = Wisconsin Card Sorting Test; RCT = Randomised Controlled Trial

*Study population comprised individuals with bipolar disorder and comorbid obsessive–compulsive symptoms rather than primary OCD

### Narrative synthesis

When broadly looking at their face validity, four of the studies selected for review [11, 12, 19, 21] report improvements in obsessive-compulsive symptoms. However, the exclusion of data from attrited patients and failure to counterbalance by conducting appropriate sensitivity analyses hinders the generalisability of their findings. Sahraian et al. [21] argued that their intention-to-treat analysis did control for this issue. However, their failure to disclose the specifics of how they conducted this analysis further highlights questions regarding the robustness of their findings. Perhaps one of the more concerning parts of Sahraian et al. [21]’s paper is its apparent reliability, with it featuring in an extensive list of meta-analyses and systematic reviews since its publication [12, 23]. As such, its findings should be interpreted with caution, with future studies demanding more comprehensive methodological control and transparency with findings. Such methodological fragilities, particularly in small and investigator-initiated trials, likely contribute to the slow accumulation of robust evidence despite memantine’s long-standing availability.

Over the past 20 years, the literature has delivered mixed conclusions on the tolerability, idealistic dose and dosing protocol of memantine to enhance its efficacy all of which are directly relevant to its potential clinical utility rather than efficacy estimation alone. Side effects were relatively consistent across the literature (Table 2), with any presenting side-effects mostly being reported as tolerable. The most common reported symptoms across all ten studies were nausea (*n* = 4), dizziness (*n* = 4), headache (*n* = 3) and drowsiness (*n* = 2). Importantly, these tolerability considerations are clinically meaningful as they may limit dose optimisation, treatment adherence and therefore the real-world therapeutic potential of memantine in OCD populations. A likely mechanism behind these is memantine’s inhibition of NMDA receptors, causing alterations in sensory processing and vestibular function [24, 25]. It evident that those who were administered stronger doses (up to 20 mg/day) were more likely to elicit aversive side-effects [11, 12, 16, 21], so this lends to determining how we can maximise the therapeutic benefits of a memantine in-situ with minimising its side effects. Accordingly, dosing and tolerability should be viewed as potential modifiers of therapeutic potential rather than endpoints in their own right, reinforcing the need for future trials to integrate these factors within efficacy-focused study designs. Ghaleiha et al. [11] and Sahraian et al. [21] employed a slow titration administration to their study procedure. By starting on lower doses and allowing the body to gradually adjust to memantine’s effects, they hypothesised that the reported side effects would be less severe. Despite this, the literature is divergent on the tolerability of memantine, with some studies emphasising the extensive tolerability of memantine over long periods of administration [26, 27]. Here, it is important to consider the inverted u-shaped pharmacodynamics of glutamate levels post-memantine administration. Research has suggested that with lower doses, memantine may deliver increasingly beneficial glutamate levels to their optimum efficacy before declining again [28]. So by considering this (and the tolerability of memantine), it could be argued that Aboujaoude et al. [16], Ghaleiha et al. [11]; Modarresi et al. [12] and Sahraian et al. [21] all administered excessive doses, increasing the prevalence of side-effects without additional therapeutic benefits. Further evidence to support his comes from another three of the studies, which, when administering 10 mg/day, found no main effects of memantine on group [17, 18, 20], alongside more minimal side effects. These findings suggest that dosing may influence both tolerability and observed outcomes; however, current evidence is insufficient to define an optimal therapeutic dose. The absence of standardised dosing frameworks across studies further complicates the comparison of findings and may have impeded the progression to larger, more confirmatory trials.

**Table 2:** Reported adverse effects and dosing are presented as described in original publications

Study (Author, Year)	% of Participants that Reported Side-Effects	Description of Side-Effects	Maximum Dosage of Memantine
Aboujaoude et al. 2009 [16]	Not reported	Side-effects were mild and transient, and no subject withdrew from the study for an adverse event.	20 mg/day
Askari et al. 2022 [17]	Not reported	Memantine showed acceptable safety and tolerability.	20 mg/day
Farnia et al. 2018 [18]	27.27% of participants in the memantine condition reported side effects	Of the three conditions, memantine was more likely to elicit rash, drowsiness, anxiety and confusion.	10 mg/day
Feusner et al. 2009 [3]	52.94% of participants reported side effects across both groups	Memantine was well-tolerated and there were no serious side effects. Side-effects experienced were dizziness and drowsiness.	20 mg/day
Ghaleiha et al. 2013 [11]	31% of patients reported a side-effect.	Frequency of side-effects between placebo and memantine was not significant. Prevalence of side-effect was more than other studies.	20 mg/day
Haghighi et al. 2013 [19]	Total % not reported; however, 2% of patients who dropped out apparently did so because of side-effects.	Measure of side effects included confusion, dizziness, drowsiness, headache, insomnia, agitation and/or hallucinations, vomiting, light-headedness, vertigo, and anxiety.	5-10 mg/day
Mirzazadeh et al. 2025 [20]	6.7% (*n* = 4) reported side effects in the memantine group.	All reported side effects were gastrointestinal. There were no reports of headache, vertigo, sleepiness, or dermatological complaints in either group.	10 mg/day
Modarresi et al. 2017 [12]	Only 13.3% (*n* = 2) of patients experienced side effects.	No difference between the two groups. Side effects reported were headache, constipation, nausea and dizziness.	20 mg/day
Sahraian et al. 2017 [21]	Three (15.8%) of people experienced side effects.	Side effects included dizziness, nausea, insomnia and headache,	20 mg/day
Stewart et al. 2010 [22]	Not reported	Not reported	18 mg/day

NB - Percentages are reported where available; several studies did not provide quantitative adverse event rates

Furthermore, the efficacy of memantine with treatment-refractory OCD is still largely unknown. Aboujaoude et al. [16], Askari et al. [17], Haghighi et al. [19] and Modarresi et al. [12] all recruited patients who had had previously been unsuccessful with a course of a SSRI, however, it is critical to note that by definition, ‘treatment-refractory’ OCD requires the individual to have been unsuccessful with at least two trials of different treatment modalities [29]. As an excellently blinded and placebo-controlled study, Modarresi et al. [12] was the only paper to effectively capture this target population (requiring three failed trials), and thus found that individuals in the memantine group elicited significantly lower Yale-Brown Obsessive Compulsive Scale (Y-BOCS) scores compared to those in the placebo (*p* = < 0.001) with 73.3% of these patients achieving a full treatment response. However, these findings should be interpreted cautiously given the limited number of trials meeting strict definitions of ‘treatment resistance’. One reason for the discrepancy of findings between the papers above and the works of Farnia et al. [18], for example, due to its shorter study length. By only administering memantine to patients for eight weeks, it is unlikely that it was capable of blocking the patients’ NMDA receptors sufficiently enough, suggesting that a more extensive time period may be needed for a significant effect to be seen.

Of the studies that found a successful therapeutic outcome via a 25% reduction in Y-BOCS scores, it is essential to consider whether baseline symptom severity influenced treatment outcomes. Across studies, inclusion thresholds for OCD severity varied substantially, ranging from Y-BOCS scores > 15 [18] to > 24 [12]. Existing literature suggests that greater baseline severity is generally associated with poorer treatment response [22, 30], which may partially account for variability in reported outcomes across trials. However, baseline severity was not consistently examined as a moderator of treatment response, limiting conclusions regarding its role in predicting memantine efficacy. Furthermore, non-response in more severe-at-baseline populations may also reflect psychosocial and contextual factors not captured within trial designs and, as with earlier trials, small sample sizes and short follow-up duration limit the extent to which these findings can be generalised.

### Updated evidence

Since the completion of the original systematic search conducted in March of 2024, we found one additional study that met our inclusion criteria and provides supplementary insight specifically into the role of memantine combined with escitalopram in treating executive function in individuals with OCD. Whilst highlighting significant improvements in OCD symptoms and highlighting minimal adverse side effects, memantine failed to elicit any significant advantages in executive function over escitalopram alone except improvements in the time management domain. Whilst enhanced time management has been proposed as beneficial for managing OCD symptomology [31], future research is needed to investigate this relationship and how memantine may mediate it. Moreover, the small sample size and restricted study duration once again significantly limit the generalisability of these findings to the broader OCD population.

### Limitations

This review has several limitations. Firstly, no formal quality assessment was undertaken. The aim of this review was to provide a descriptive overview of the current breadth of research available and explore emerging themes (such as side effects and tolerability) rather than solely the efficacy of memantine as an intervention. The number of studies included in the review was also relatively small, however this is due to memantine being an off-label treatment with a limited evidence base. Resultantly, only a limited number of rigorous RCTs were available for review. Furthermore, substantial heterogeneity across study designs, populations, dosing protocols, and outcome measures precluded meta-analysis, necessitating a narrative synthesis. While this limits quantitative comparison across studies, it allowed for detailed critical appraisal of methodological features influencing interpretation of reported outcomes.

A notable limitation of the analysed evidence base is the strong geographical concentration of included RCTs, with the majority conducted in Middle Eastern research settings. There is no single established explanation for the geographical concentration of studies and as such we have framed this discussion cautiously, highlighting potential contributing factors without attributing causality. This clustering may reflect regional research priorities, prescribing practices, and healthcare infrastructures, and raises critical questions regarding the external validity and generalisability of findings to other populations and clinical contexts. It is likely that differences in diagnostic pathways, treatment availability, and background pharmacological exposure may influence both baseline characteristics and treatment response and should be considered when interpreting results across studies. Because of this, the future replication of findings in more diverse geographical and healthcare settings is required to establish the broader applicability of memantine as a potential adjunctive treatment for OCD.

Despite the long-standing availability and established use of memantine in other neurological conditions, its investigation in OCD has remained relatively limited. This may be secondary to challenges inherent to conducting pharmacological trials in treatment-refractory OCD populations, including difficulties in recruitment, heterogeneous clinical presentations, and variability in outcome measures. Furthermore, inconsistent dosing strategies and short study durations across previous RCTs may have limited confidence in reported findings, reducing any progression to subsequent large-scale investigations. As a repurposed medication with limited commercial incentive, memantine has also been less likely to attract sustained industry-driven research, underscoring the need for independent, methodologically robust studies to clarify its therapeutic role in managing OCD.

Overall, these limitations highlight not only the constraints of the present review but also broader structural challenges within existing literature that has limited the cumulation of evidence in this under researched area.

### Future directions

Future research should aim to prioritise large sampled, high quality RCTs to further our understanding of the efficacy and tolerability of memantine as an adjunctive treatment for obsessive-compulsive disorder. Further, standardised outcome measures and consistent diagnostic criteria across all future studies will allow for better comparison and meta-analytic syntheses in future systematic and literature reviews. Extending future studies with longer follow-up periods will also be invaluable in assessing the long-term effects of memantine in a population of individuals with OCD. Finally, the inclusion of neurobiological and/or biomarker data in future larger-scale studies could also help us to elucidate memantine’s mechanism of action in treating obsessive-compulsive disorder.

## Conclusion

Given methodological heterogeneity, limited sample sizes, and inconsistent definitions of treatment resistance, memantine cannot currently be recommended for routine clinical use in OCD. While some trials report symptomatic improvement in narrowly defined treatment-refractory populations, these findings require cautious interpretation. Overall, the existing literature highlights the need for larger, methodologically robust randomised controlled trials with standardised dosing strategies and longer follow-up periods that clarify memantine’s tolerability and therapeutic potential. Accordingly, the added value of this review lies in critical appraisal of study design, dosing, tolerability, and methodological limitations, rather than a pooled efficacy estimation.

## Data Availability

No datasets were generated or analysed during the current study.
